# Experimental Cholecystotomy

**Published:** 1884-10

**Authors:** J. McF. Gaston

**Affiliations:** Atlanta, Ga.


THE ATLANTA

MEDICAL AND SURGICAL JOURNAL.

VOL. I. OCTOBER, 1884. No. 8.

### Original Communications.

### EXPERIMENTAL CHOLECYSTOLOMY.

BY J. McF. GASTON, M. D., ATLANTA, GA.

[*Concluded.*]

August 19th, 1884--Case No. 2, upon which my calculations were based for the most satisfactory result, escaped last night, and a notice has been put in the afternoon journal offering a reward for the animal, but I fear nothing will come of it.

The wounds of Nos. 1 and 5 are favorable, excepting that several stitches are detached in the latter; so that after replacing them, with his mouth muzzled, the dog was suspended by placing a strong piece of cotton drill beneath his body, which extended from the shoulders to the quarters, and with straps around the front to the rear of these parts respectively. The four extremities were then secured above the back of the animal to the cords that were tied to a beam in the low roof of the shed, at such elevation as to keep the dog's feet off the ground. Another cord was fastened to the head strap of the muzzle, while still another secured to the rump band held up these parts effectually and with little discomfort to the dog.

August 20th, 1884—The nose band of the muzzle on the suspended dog has caused considerable swelling of the lip and nose so that it is removed, but the animal remains in his elevated support. Having neither drank nor eaten anything since yesterday morning, it was remarkable that he did not care for water, and yet ate raw beef freely.

On the afternoon of this day a female puppy of the small breed of dogs was operated upon in the presence of two colleagues, Drs. Jas. A. Gray and W. G. Owen. This was etherized, and an incision not exceeding two and a half inches enabled me to pass a silk ligature of doubled thread through a very small portion of the wall of the gall-bladder and also through less than the fourth of an inch of the duodenal wall by which they were bound together. The same thread, single, was used to close the peritoneal incisions by continuous suture, and the external cutaneous incision by interrupted suture in No. 6.

There was ooz ng of bile-stained serum from the wound that night and next morning, but it ceased from that time.

August 23d, 1884—Case No 1 was reported to have died about noon and the autopsy was made with the presence of Drs. Divine and Kerstan, revealing the fact that although the external incision was not closed at all points, yet the peritoneal cut was united throughout. Upon dividing the tissues I found the continuous ligature embedded in them, and still so strong as to resist a considerable strain upon the pieces that were drawn out; and this after the lapse of fourteen days since the operation. On entering the cavity there was considerable discharge of a decomposed serous fluid or effusion from the extensive peritoneal inflammation, and in exploring further a large abscess was found at the posterior hypochondriac region in which the substance of the liver was involved. There were extensive adhesions throughout the abdominal cavity, and had evidently been accompanied with enlargement and degeneration of the gall-bladder, so that it had become the medium of agglutination between different parts of the liver.

In separating them the cavity of the gall bladder was exposed and the well-knotted elastic ligature was found within it near a fistulous opening into the duodenum. It may be stated in passing that the elasticity of the ligature was well preserved, and the fact of its falling into the gall-bladder instead of dropping through into the canal may have resulted from the wall of the former giving way earlier than that of the latter, and calls for some means to be adopted for its prevention in future. The small slit opening from the duodenum into the gall-bladder illustrates the principle of action on the part of the ligature most satisfactorily, while the close adhesion around this orifice proves the efficacy of the continuous circular catgut stitches. I have preserved this specimen in alcohol, so that all who are interested to examine the progress made towards solving the practicability of effecting an artificial communication between the gall-bladder and the upper intestinal canal, may see it.

August 29th, 1884—After the mouth of No. 5 was set free from the muzzle, it was not practicable to prevent interference with the suspensory apparatus, and on the following day one of the cords had been cut with the teeth so that the animal was resting upon his feet, but as all seemed still secure around the body, no change was made. Upon visiting my patient next morning, it was discovered that some of the nose fastenings had been torn loose, so that the supporting belt was escaping from behind and new attachments were secured around the quarters of the animal. He now had some play upon the ropes so as to walk around a small circle, and being without the muzzle, doubtless set to work with his teeth so that on yesterday morning the suspensory belt was relieved entirely from behind, and was doubled up around the thorax so as to expose the wound from which the stitches were torn out.

As there was no protrusion of either intestine or omentum it was thought sufficient to put a compress over the wound, and drawing the bandage or belt well back to the inguinal and pubic region, it was sewed to straps that passed back and around the quarters so as not to escape forward. This was done, leaving the animal on its feet and with the mouth free; and this morning he was reported to me as having torn loose all the fastenings anteriorly so that the wound was exposed and a great part of the intestines with the spleen were upon the ground, while the gravest apprehensions were entertained as to life. Death soon occurred, and on making the post mortem examination, adhesions were found of various parts adjacent to the liver, while there was disorganization of the walls of the gall-bladder in places, and yet with most complete adhesion of the surface of the duodenum to its lower surface, and a well defined fistulous opening between the cavity of the duodenum and the gall-bladder.

This opening, that resulted from the cutting of the elastic ligature, had in the present case allowed of its escape into the canal, differing in this respect from the previous case in which the ligature was retained in the cavity of the gall-bladder. The orifice through the adherent walls had a portion of the mucous membrane of the intestine reflected over the margin of the inner surface of the gall-bladder, and gave a striking correspondence to the exterior of the lining membrane of the mouth to the lips in the human race. The slit was very much like a little mouth, with the lips partially open in the middle, and presents a perfect type of a fistulous opening. In so far as the adherence of the walls attached by the circular continuous suture around the elastic ligature indicates success, nothing was wanting, and the manipulation requisite to let the ligature drop into the duodenal canal is now perhaps the most essential thing to be determined.

It is probable that there was greater pressure of the knot towards the wall of the gall-bladder or that of the duodenum in the respective case of the ligature dropping in either direction. This tendency to go towards the cavity into which the knot may first cut its way is in accordance with the simplest mechanical principles, and the entrance of the ligature through the artificial opening into the duodenum is likely to be secured by pressing the knot towards its wall at the close of the ligature.

In a suture of an incision through the wall of the intestine alone, there is a layer of plastic lymph thrown out that covers the outer surface in such a manner as to cause the loop to make its way into the cavity of the canal, but no similar process operates in uniting the wall of the duodenum with that of the gall-bladder so far as the two cavities are concerned, and hence the knot pressure gives direction to the loop.

On the 28th of September, 1884, case No. 6 was etherized and with the assistance of Dr. W. S. Elkin, the line of the former incision was laid open, and it was verified that the adhesion of the wall of the duodenum to that of the gall-bladder was complete at the point where the single silk ligature had been used to unite them. It was also inferred from the small quantity of bile in the gall-bladder that the opening between them was fully established, but as the demonstration of this fact beyond any kind of doubt was only practicable by laying open one or the other of the cavities, and thus most likely causing the death of the animal, it was not resorted to. I had intended, if practicable on this occasion, to ligate the cystic duct in the ductus choledochus, but the adhesions of the wall of the duodenum over the line of the duct prevented this being done, and the main point being established, the wound was closed with the hairlip suture of needles and thread, so crossing as to keep the borders in apposition.

I had been in some doubt, after this experiment was made, on the 20th, about the parts being held together by the silk ligature, but the examination satisfied both of us, not only that the ligature had united the exterior surfaces, but that it must have opened the communication between the cavity of the gall bladder and that of the duodenal canal. Thus complete success has attended this mode of proceeding without any material impairment of the health of the animal and no extension of inflammation to the abdominal viscera.

In this case a jacket of cotton cloth was sewed around the body of the animal and strips secured it around the breast and around the quarters so that there could be no slipping forward or backward, and this was not removed for a week. The stitches were then found loosened by cutting the external integument, but the peritoneal suture had done its work satisfactorily, and the closure was completed at the date of re-opening the cavity of the abdomen.

On the 1st of September, 1884, the jacket of case No. 6 was removed and the pins were found to have cut their way out, but there was union of the inner layer of tissues, and it was not found necessary to use any means of approximating the gaping integument.

A compress of old soft cotton cloth soaked in carbolized oil was bound over the wound and the jacket re-applied.

September 5th, 1884.—The animal eats and moves about the room, leading to the inference that the result will be favorable, and my intention is to keep this case for some months to verify the continuity of the opening between the gall-bladder and the duodenum.

This satisfactory solution of the problem for restoring the bile to the alimentary canal in place of letting it escape by an external fistulous opening, only remains to be tested in the human subject.

